# Prenatal Influence and Race Betterment

**Published:** 1914-08

**Authors:** 


### Prenatal Influence and Race Betterment.

While an inherent hereditary character cannot be changed by any influence operating on it, in course of its development in embryo, yet it is plainly seen how abnormal agencies, acting through the mother, could affect the quality of the character. Just as environment of the growing child can be made to suppress evil tendencies, altering the natural course of the child's predisposition, so can conditions affecting his maternal environments, in a still greater measure, alter the development of the embryo because of its greater plasticity. A plant of a certain species, if cultivated under normal conditions, will grow and produce the qualities usual to that species, but if it is cultivated under adverse conditions, while it will produce the characteristics (fruits) expected, they will be of an inferior quality. The embryo and fetus is the field in which the human species is planted and developed. The inherent qualities contained in the germinal cells determine whether the seed will germinate wheat or corn and the specific species of the wheat or corn; but the maternal environment—representing the soil and cultivation of the germ cell—determines whether the wheat or corn, as it may be, will produce its best quality.

Thus we see how environment is absolutely dependent upon eugenics—nurture on nature—and how, on the other hand, eugenics is dependent upon environment. This being true, the most tractable period for operation in the betterment of the child is during the period of gestation. It is then that the whole system undergoes a decided physiological change to meet the extra demands made upon it. The blood is more abundant and richer, the liver is greatly enlarged to perform its important function of incineration. The heart must perform a greater work; consequently, its muscles adapt themselves to cope with the new emergency. So systematic and intricate are all these functions involved in the development of the embryo that any abnormality of any one will have a corresponding effect on all the others with its consequent effect on the embryo. Therefore, any attempted betterment of the race that does not consider the maternal environment will fail in its essential purpose.

The child born right has a much better chance to adapt himself to his environment than one who is born with the handicaps that come through bad heredity and improper care during gestation. Leaving aside the strictly hereditary characters, which we do not yet know how to control, much can be done through gestational therapeutics. We recognize the importance of fresh air in oxidizing the impurities of the blood, and the necessity of nourishment to meet the demands of metabolism and to provide food for the growing embryonic tissues. We realize how necessary the kidneys are in eliminating poisonous substances; the liver in destroying the products of embryonic digestion; how the womb must be healthy to furnish proper soil for the development of the placenta. And, more than this, we also know how psychic influences affect the functions of the organs and how certain racial poisons, so-called, even affect the character (quality) of the germinal cells, modifying their character that the child so born may be said to have a vitiated constitution.

This involves the right of the mother who is not physically strong and whose environment is such as not to afford the best opportunity for motherhood to give birth to a child, and an added duty on the physician to medicinally treat every woman affected with disease during the period of gestation.

Obviously, then, the duty of race betterment falls upon both the eugenist (biologist) and the obstetrician and, incidentally, on the social control that demands a larger measure of service on the part of the obstetrician and unhampered license to the work of the legitimate biologist.

---

The Hood-Somervell County Medical Society is certainly a live one, and some of the presidents of county societies who find it hard work to get the doctors together these hot days would do well to profit by their example. At the mid-summer meeting, which falls to Glen Rose, they have been having basket picnics for the doctors and their families. This year they invited the doctors in the adjoining counties and the general public.

To make the meeting interesting to all who may attend, a Better Baby Contest is held, and the physicians speak on subjects that are interesting and helpful to the general public. The members of Hood-Somervell County Medical Society are as follows: Dr. J. R. Lancaster, Granbury; Dr. E. S. Menefee, Granbury; Dr. J. A. Jarrett, Granbury; Dr. E. H. Morgan, Granbury; Dr. T. H. Dabney, Granbury; Dr. G. N. Lancaster, Granbury; Dr. J. M. McCuin, Mambrino; Dr. J. W. McFall, Lipan; Dr. J. H. Gandy, Lipan; Dr. F. A. Pollins, Acton; Dr. A. B. McCallon, Carson; Dr. E. C. Price, Tolar; Dr. J. D. Ross, Paluxy; Dr. W. B. Prewitt, Georges Creek; Dr. H. Wilder, Glen Rose; Dr. A. B. Currie, Glen Rose; Dr. W. H. Powell, Glen Rose; Dr. L. P. Gibbs, Glen Rose; Dr. E. A. Milam, Glen Rose.

